# INTERGENERATIONAL BONDS ACROSS THE EARLY YEARS OF MARRIAGE

**DOI:** 10.1093/geroni/igad104.1742

**Published:** 2023-12-21

**Authors:** Raquael Joiner, Jacqueline Perez, Thomas Bradbury, Benjamin Karney

**Affiliations:** University of Southern California , Los Angeles, California, United States; University of California, Los Angeles, Los Angeles, California, United States; University of California, Los Angeles, Los Angeles, California, United States; University of California, Los Angeles, Los Angeles, California, United States

## Abstract

As adults embark upon marriage, forming their own new nuclear families, they often remain connected to their families of origin. Nevertheless, spouses’ relationships with their parents and in-laws are likely to change across the early years of marriage. Given that empirical studies to date are typically limited to only a couple of measurement occasions, however, the stability and impact of such changes are unclear. Using Repeated Measures Latent Class Analyses, the present study addresses this gap by identifying classes of spouses (N=375 dyads) experiencing different types of relationships with their parents and in-laws across the first 9 years of marriage. Further, correlates and consequences of spouses’ longitudinal sentiment profiles were also examined. Three sentiment profiles (i.e., Mostly Helpful, Mostly Ambivalent, Mostly Indifferent, Mostly Difficult) emerged when looking at spouse’s sentiments toward their own parents and for husbands’ sentiments towards their in-laws, whereas 4 profiles (i.e., Mostly Helpful, Mostly Ambivalent, Mostly Indifferent, Mostly Difficult) were found for wives’ sentiments towards their in-laws. Mostly Ambivalent sentiment profiles showed the least stability, and spouses showed significantly higher odds of being in the Mostly Ambivalent class if they were living in a multigenerational household and if the wife was a 2nd generation immigrant. Spouses whose marriages dissolved showed significantly higher odds of being in the Mostly Indifferent, Mostly Ambivalent, and Mostly Difficult classes, compared to the Mostly Helpful class. These results provide an important starting point for future research aiming to understand how intergenerational bonds may promote or hinder successful marriages.

